# Quaternion wavelet transform based full reference image quality assessment for multiply distorted images

**DOI:** 10.1371/journal.pone.0199430

**Published:** 2018-06-27

**Authors:** Chaofeng Li, Yifan Li, Yunhao Yuan, Xiaojun Wu, Qingbing Sang

**Affiliations:** 1 Institute of Logistics Science & Engineering, Shanghai Maritime University, Shanghai, China; 2 School of Internet of Things Engineering, Jiangnan University, Wuxi, China; 3 College of Information Engineering, Yangzhou University, Yangzhou, China; Beijing University of Technology, CHINA

## Abstract

Most of real-world image distortions are multiply distortion rather than single distortion. To address this issue, in this paper we propose a quaternion wavelet transform (QWT) based full reference image quality assessment (FR IQA) metric for multiply distorted images, which jointly considers the local similarity of phase and magnitude of each subband via QWT. Firstly, the reference images and distorted images are decomposed by QWT, and then the similarity of amplitude and phase are calculated on each subband, thirdly the IQA metric is constructed by the weighting method considering human visual system (HVS) characteristics, and lastly the scores of each subband are averaged to get the quality score of test image. Experimental results show that the proposed method outperforms the state of art in multiply distorted IQA.

## Introduction

With the large-scale use of intelligent mobile phone and computer in our modern society, evaluating the images after compression and transmission has become an increasingly important issue, and image quality assessment (IQA) has the great practical significance.

IQA can be divided into three types: full-reference IQA, reduced-reference IQA, and no-reference IQA. The full reference IQA is developed earliest, which uses the original image as a reference. Full reference IQA can be roughly divided into error visibility and structural similarity methods. Peak signal-to-nosie (PSNR) is the simplest full reference IQA method. The structural similarity (SSIM) [1] index was proposed base on the human visual system (HVS) characteristics. Also, Wang et al. [2] further proposed a multi-scale version of SSIM, called MS-SSIM. Zhang et al. [3] proposed a feature similarity (FSIM) index that uses phase congruency to weight the quality score based on SSIM index. Kolaman et al. [4] used a quaternion matrix to express a color image, and then calculated the structural similarity to evaluate color image quality. Liu et al. [5] proposed an IQA scheme based on the concept of gradient similarity (GSIM) to alleviate the shortcoming of relevant schemes. Wu et al. [6] integrated the merits of existing IQA metrics with the guide of internal generative mechanism (IGM). Xue et al. [7] devised a full reference IQA model called gradient magnitude similarity deviation (GMSD). Saad et al. [8] presented a no-reference IQA algorithm named BLIIND-II based on a natural scene statistics model of discrete cosine transform (DCT) coefficients.

In recent years, quaternion wavelet transform (QWT) has been widely used in image processing. For example, Chen et al. [9] used hybrid phase congruency extracted by QWT and gradient magnitude to calculate the similarity of images. Traoré et al. [10] proposed a reduced-reference metric based on QWT coefficients and confirmed that QWT produces a better coefficient of correlation with HVS than discrete wavelet transform (DWT). Tang et al. [11] proposed a novel dual-tree QWT based blind camera image quality assessment metric.

Motivated by recent progress in IQA and QWT, in this paper we propose a QWT based full-reference IQA metric called QWT-IQA, which jointly takes into account the local similarity of phase and magnitude of each subband via quaternionic wavelet transform. In our QWT-IQA, we make use of a weighting method to compute the image quality score, inspired by human visual characteristics. A lot of experimental results demonstrate that our proposed QWT-IQA outperforms existing full-reference IQA method.

The remaining part of the paper is organized as follows. In the section Background, we introduce the quaternion and QWT. In the section QWT-based full reference IQA metric, we propose a full-reference IQA metric based on QWT. Experiments on the LIVEMD and MDID2013 databases are carried out in the section Experimental results and analysis. At last, we give the conclusion in the section Conclusion.

## Background

### Review on quaternion

The quaternion is a mathematical concept proposed by an English mathematician in 1843. We all know real number and imaginary number in mathematics; and quaternion is an expansion of imaginary number. The imaginary number has a real part and an imaginary part, and the quaternion has one real part and three imaginary parts similarly. If *q* is a quaternion, it can be expressed as [12]: *q* = *a*+*bi*+*cj*+*dk*, where *a*, *b*, *c*, and *d* are the real numbers, *a* is the real part of quaternion, and *bi*+*cj*+*dk* is the imaginary part of quaternion, and *i*, *j*, and *k* are imaginary numbers satisfying the following

$$\left\{ \begin{array}{l} i^{2}=j^{2}=k^{2}=-1\\ ij=-ji=k,jk=-kj=i,ik=-ki=j \end{array}\right.$$ (1)

A quaternion can also be expressed by amplitude and phase, i.e., *q* = |*q*|*e*^*iφ*^*e*^*jθ*^*e*^*kψ*^, where |*q*| is the amplitude, and *φ*,*θ*,*ψ* are the phase angles whose range are [−*π*,*π*], [−*π*/2,*π*/2], and [−*π*/4,*π*/4] respectively.

### Review on QWT

Quaternion wavelet transform is a new wavelet transform, which combines quaternion and Hilbert transform together. It is approximately shift-invariant and has abundant phase information. The four orthonormal bases of QWT can be expressed in the matrix form as follow.

$$G=\left[\begin{array}{cccc} \varphi_{h}\left(x\right)\varphi_{h}\left(y\right) & \varphi_{h}\left(x\right)\psi_{h}\left(y\right) & \psi_{h}\left(x\right)\varphi_{h}\left(y\right) & \psi_{h}\left(x\right)\psi_{h}\left(y\right)\\ \varphi_{g}\left(x\right)\varphi_{h}\left(y\right) & \varphi_{g}\left(x\right)\psi_{h}\left(y\right) & \psi_{g}\left(x\right)\varphi_{h}\left(y\right) & \psi_{g}\left(x\right)\psi_{h}\left(y\right)\\ \varphi_{h}\left(x\right)\varphi_{g}\left(y\right) & \varphi_{h}\left(x\right)\psi_{g}\left(y\right) & \psi_{h}\left(x\right)\varphi_{g}\left(y\right) & \psi_{h}\left(x\right)\psi_{g}\left(y\right)\\ \varphi_{g}\left(x\right)\varphi_{g}\left(y\right) & \varphi_{g}\left(x\right)\psi_{g}\left(y\right) & \psi_{g}\left(x\right)\varphi_{g}\left(y\right) & \psi_{g}\left(x\right)\psi_{g}\left(y\right) \end{array}\right]$$ (2)

where *φ*_*h*_ and *φ*_*g*_ are scale functions, *ψ*_*h*_ and *ψ*_*g*_ are wavelet functions. According to [13], each row of the matrix *G* in (2) represents the independent wavelet of QWT, and each column of the matrix represents a subband of QWT. By using the algebra about quaternion, the four groups in each column can be grouped into a wavelet function

$$\psi^{q}\left(x,y\right)=\psi_{h}\left(x\right)\psi_{h}\left(y\right)+i\psi_{g}\left(x\right)\psi_{h}\left(y\right)+j\psi_{h}\left(x\right)\psi_{g}\left(y\right)+k\psi_{g}\left(x\right)\psi_{g}\left(y\right)$$ (3)

## QWT-based full reference IQA metric

**Fig 1:**
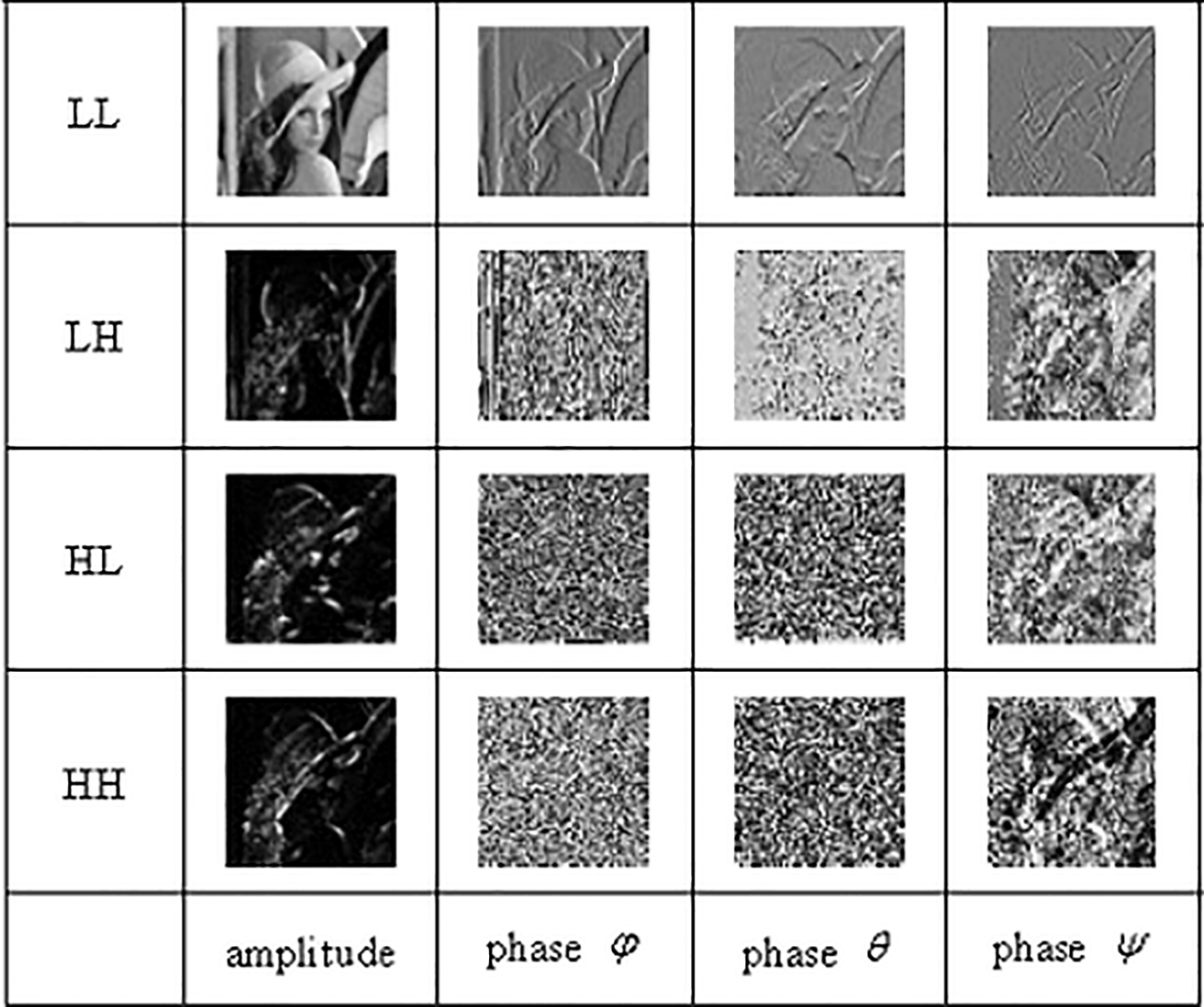
Amplitude and phase images of each subband via QWT.

As above, QWT is a new dual-tree wavelet that has shift invariance and phase information. In this work, based on the local similarity of phase and magnitude of each subband via QWT, we construct a FR IQA metric. Firstly, the reference image and corresponding distorted image are, respectively, decomposed into a low frequency subband (LL) and three high frequency subbands (LH, HL, HH) at each scale via QWT. Then, an amplitude and three phases are got at each subband. Through a large number of experiments, we acquire the best performance when the image is decomposed by 3 scales QWT. The amplitude of low frequency subband reflects the condition of the original image and has approximate shift invariance. The phases (*φ*,*θ*,*ψ*) of low frequency subband indicate the vertical, horizontal and diagonal texture information, respectively. The amplitude of high frequency subband reflects the contour of the image in some particular direction. The phases (*φ*,*θ*) of high frequency represent local shift information, and the phase *ψ* of high frequency obtains the texture feature of image. Fig 1 shows the images of amplitude and phase (*φ*,*θ*,*ψ*) of each subband via QWT.

**Fig 2:**
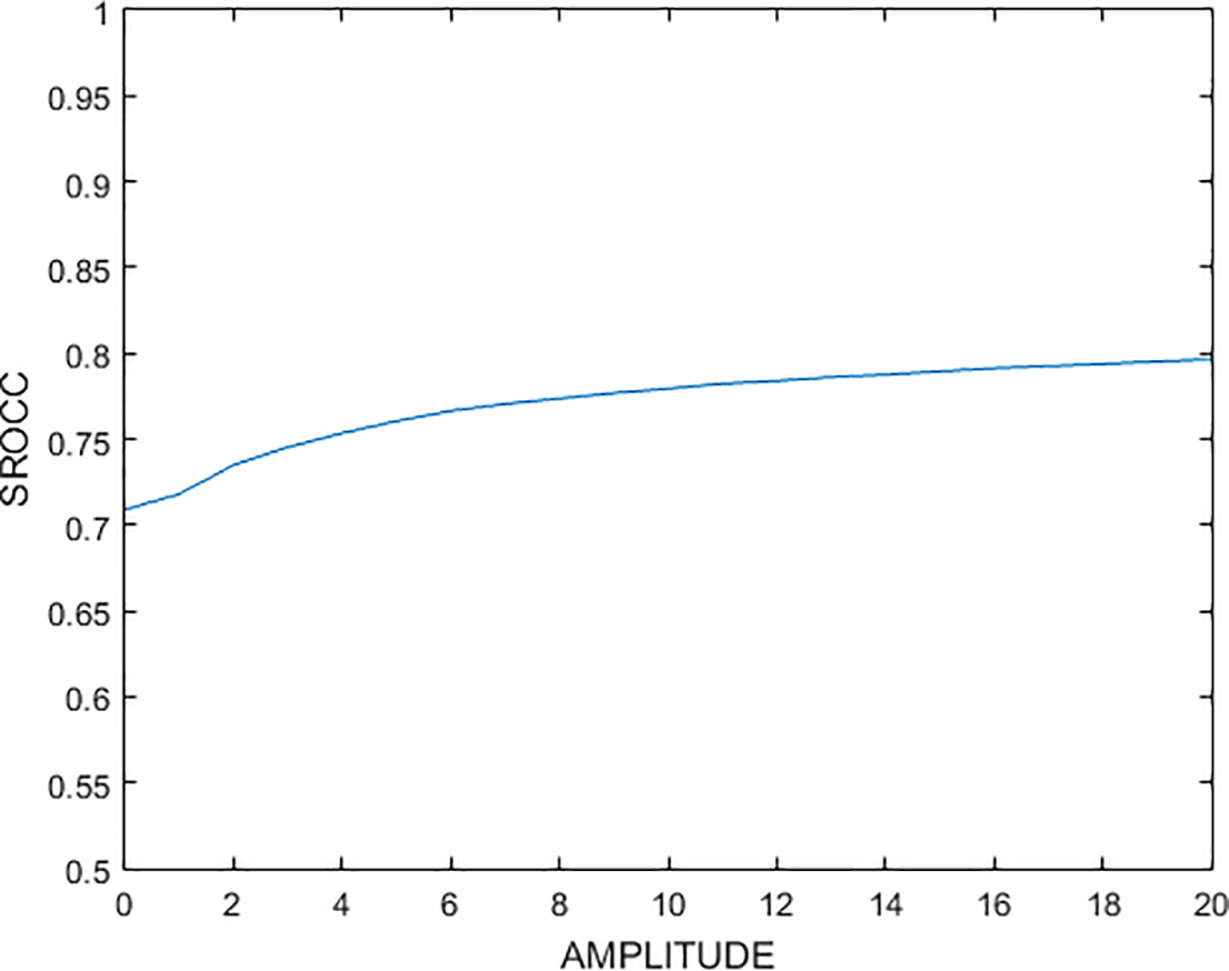
The plot between SROCC and amplitude.

Now, we calculate the similarity of magnitude and three phases of the reference image and distorted image. The similarity of magnitude is defined as

$$S_{Mag}\left(x\right)=\frac{2Mag_{1}Mag_{2}+T_{1}}{Mag_{1}{}^{2}+Mag_{2}{}^{2}+T_{1}}$$ (4)

where *Mag*_1_ and *Mag*_2_ represent the amplitude of LL subband of the original image and corresponding distorted images by 3-scale QWT, and *T*_1_ is a positive normal number, which aims to make the denominator non zero. Following the same way as (4), the similarities of phase (*φ*,*θ*,*ψ*) are defined by

$$S_{\varphi }\left(x\right)=\frac{2\varphi_{1}\varphi_{2}+T_{2}}{\varphi_{1}{}^{2}+\varphi_{2}{}^{2}+T_{2}}$$ (5)

$$S_{\theta }\left(x\right)=\frac{2\theta_{1}\theta_{2}+T_{3}}{\theta_{1}{}^{2}+\theta_{2}{}^{2}+T_{3}}$$ (6)

$$S_{\psi }\left(x\right)=\frac{2\psi_{1}\psi_{2}+T_{4}}{\psi_{1}{}^{2}+\psi_{2}{}^{2}+T_{4}}$$ (7)

where *φ*_1_,*θ*_1_,*ψ*_1_ and *φ*_2_,*θ*_2_,*ψ*_2_ represent phases of LL subband of the original image and distorted images, and *T*_2_,*T*_3_,*T*_4_ are positive normal numbers, which aim to maintain the fraction stability. Note that the similarity range of (5), (6) and (7) is (0,1]. The local similarity of LL subband is defined as follows:

$$S_{L}\left(x\right)={\left[S_{Mag}\left(x\right)\right]}^{\alpha }\cdot {\left[S_{\varphi }\left(x\right)\right]}^{\beta }\cdot {\left[S_{\theta }\left(x\right)\right]}^{\chi }\cdot {\left[S_{\psi }\left(x\right)\right]}^{\delta }$$ (8)

where the coefficients α, β, χ, δ represent the importance of amplitude and phases. The amplitude of low frequency subband reflects the condition of the original image, and which of high frequency subband denotes the contour of the image in some particular direction. By setting phase β, χ, δ to 1, and changing the value of magnitude α from 0 to 20 step by 1, we gain the plot between SROCC and amplitude shown in Fig 2. It can be seen the SROCC rose gently when the amplitude is set to 10, so we set the magnitude α to 10, and the phase β, χ, δ to 1, then the Eq (8) become following Eq (9).

$$S_{L}\left(x\right)=S_{Mag}^{10}\left(x\right)\cdot S_{\phi }\left(x\right)\cdot S_{\theta }\left(x\right)\cdot S_{\psi }\left(x\right)$$ (9)

After getting the similarity of each pixel, the similarity of the LL subband can be calculated. However, the human visual system has different perceptual effects on different regions of the picture. The more obvious the pixel amplitude is, the more prominent its corresponding phase is. The larger the amplitude is, the more important the corresponding pixel is, and the more likely it is to be in the texture changing structure. The smaller the amplitude is, the more likely it is to be in the smooth image region, and the value of the corresponding phase tends to be more unstable [14]. In other words, the human eyes are always devoting more attention to the area which has larger amplitude. Thus, we add the item *Mag*_*m*_(*x*) = max(*Mag*_1_,*Mag*_2_) to make the IQA metric more consistent with human visual characteristics. Therefore, the SImilarity Metric in LL subband (*SIM*_*LL*_) considering HVS is calculated as following.

$$SIM_{LL}=\frac{\sum_{x\in \Omega }S_{L}\left(x\right)\cdot Mag_{m}\left(x\right)}{\sum_{x\in \Omega }Mag_{m}\left(x\right)}$$ (10)

where Ω represents the entire space domain of the image.

Following the same computation way in (10), the SImilarity Metrics in LH, HL and HH subbands, i.e., *SIM*_*LH*_, *SIM*_*HL*_, *SIM*_*HH*_ can also been calculated in a similar form. Once the foregoing four similarity metrics are obtained, the QWT-based IQA metric (QWT-IQA) can be calculated by a weight sum of similarity metrics in all subbands as follows.

$$SIM_{LH}=\frac{\sum_{x\in \Omega }S_{L}\left(x\right)\cdot Mag_{m}\left(x\right)}{\sum_{x\in \Omega }Mag_{m}\left(x\right)}$$ (11)

$$SIM_{HL}=\frac{\sum_{x\in \Omega }S_{L}\left(x\right)\cdot Mag_{m}\left(x\right)}{\sum_{x\in \Omega }Mag_{m}\left(x\right)}$$ (12)

$$SIM_{HH}=\frac{\sum_{x\in \Omega }S_{L}\left(x\right)\cdot Mag_{m}\left(x\right)}{\sum_{x\in \Omega }Mag_{m}\left(x\right)}$$ (13)

$$QWT‑IQA_{\Omega }=a\cdot SIM_{LL}+b\cdot SIM_{LH}+c\cdot SIM_{HL}+d\cdot SIM_{HH}$$ (14)

where *a*, *b*, *c* and *d* are used to adjust the importance of each subband.

**Fig 3:**
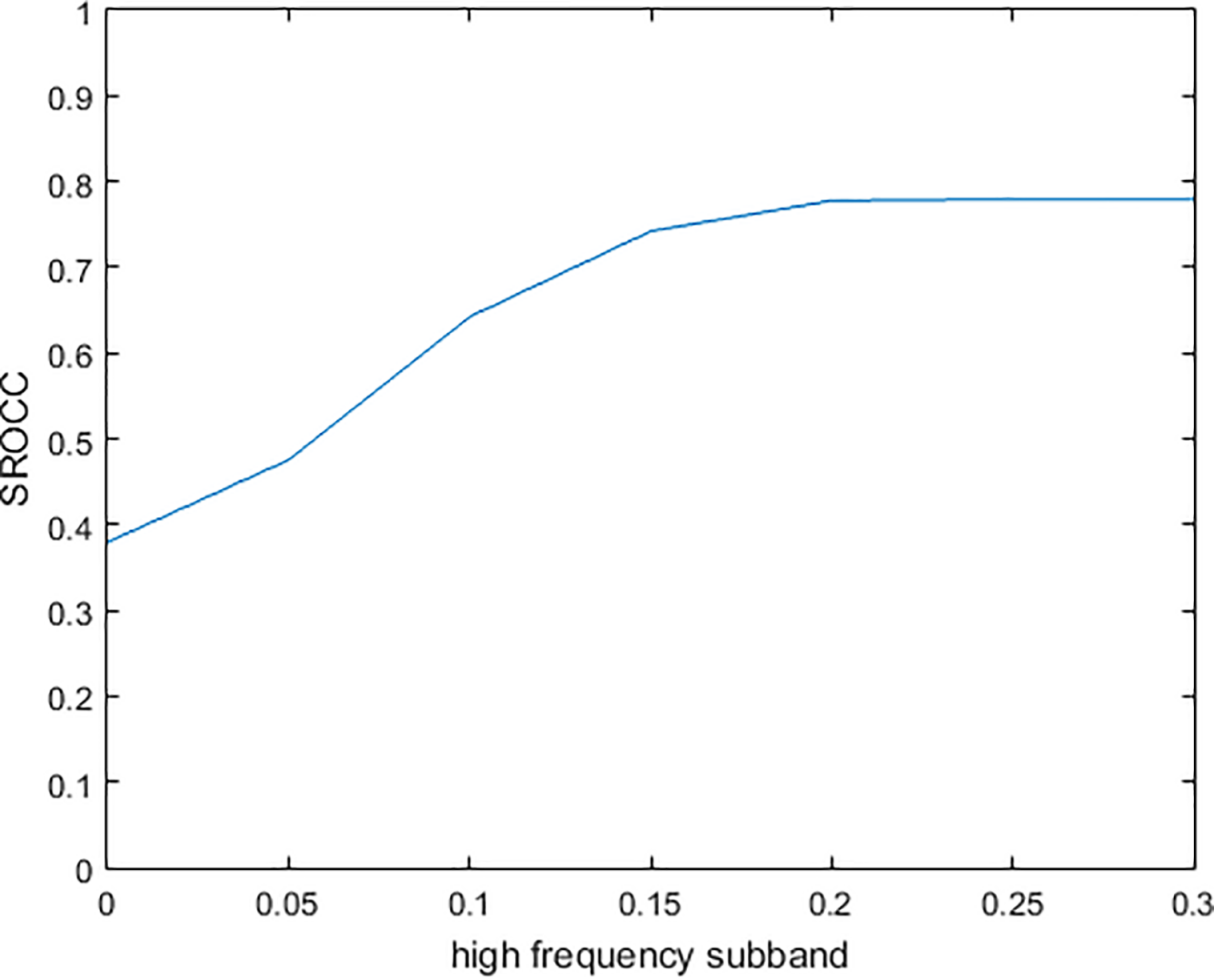
The plot between SROCC and subband coefficients.

From Fig 1 the three high frequency subbands are similar each other, so we keep them the same value, and change from 0 to 0.3 step by 0.05, then the coefficient of low frequency from 1 to 0.1 correspondingly, and gain the plot between SROCC and subband coefficients shown as Fig 3. The curve becomes smooth when the coefficients are close. It can be seen the SROCC reaches maximum when coefficients of high frequency subbands are 0.25, which suggest each subband is the same important for our proposed IQA metric.

So here we set all *a*, *b*, *c* and *d* to 0.25, and the final IQA formula is defined as follows:

$$QWT‑IQA_{\Omega }=0.25\cdot \left(SIM_{LL}+SIM_{LH}+SIM_{HL}+SIM_{HH}\right)$$ (15)

**Fig 4:**
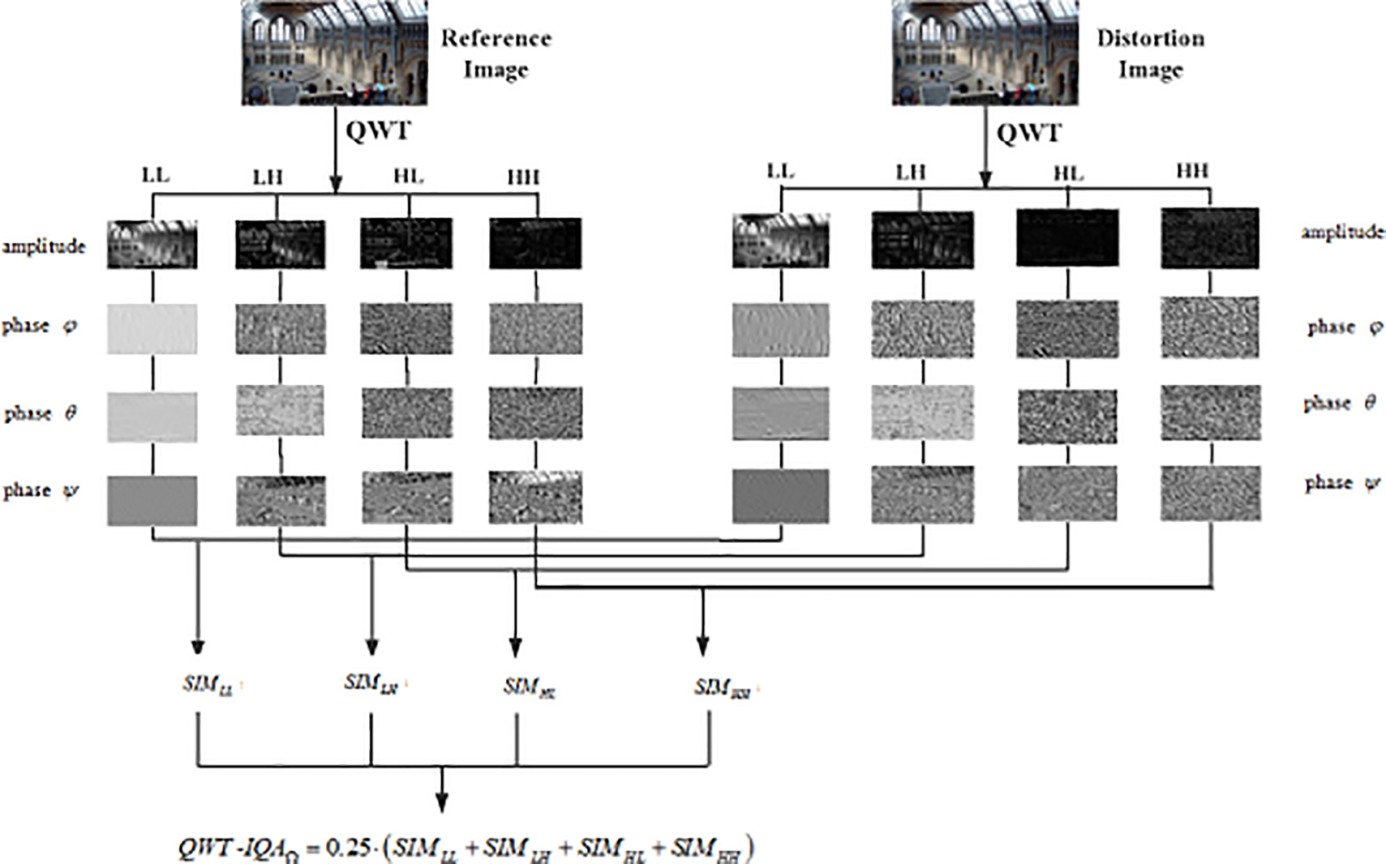
The flowchart of QWT-IQA.

The whole flow chart of our proposed QWT-IQA is illustrated in Fig 4.

## Experimental results and analysis

### Experiment on the LIVEMD image database

LIVE multiply distorted (LIVEMD) image database [15] consists of 15 reference images and 450 distorted images, which has two multiple distortion scenarios: blur followed by JPEG and blur followed by noise. Each image set has 90 singly distorted images and 135 multiply distorted images, and the size of the images is 1280×720. The difference mean opinion score (DMOS) of LIVEMD is between 0 and 100.

There are several measures to evaluate the correlation between the quality scores and DMOS, such as Spearman rank order correlation coefficient (SROCC), Pearson linear correlation coefficient (PLCC), Kendall’s rank order correlation coefficient (KROCC), and Root mean squared error (RMSE). Note that, the closer correlation coefficient is to 1 and the lower RMSE is, the better the algorithm performs.

Experiments are carried out on two image subsets and the entire database, respectively. We compare our proposed QWT-IQA with the FR PSNR, SSIM, FSIM, MS-SSIM and NR SISBLIM, DIIVINE, BLIINDS-II, NIQE methods. The results are listed in Tables 1, 2 and 3. From Tables 1, 2 and 3 it can be seen that QWT-IQA performs better than these FR and NR algorithms on both image subsets and whole database, no matter what measure is used.

**Table 1:** Several IQA algorithm comparison on the blur and JPEG image dataset.

IQA metrics	Type	SROCC	PLCC	KROCC	RMSE
PSNR	FR	0.6621	0.7409	0.4775	12.8696
SSIM	FR	0.7443	0.8003	0.5430	11.4895
FSIM	FR	0.8546	0.9065	0.6606	8.0892
MS-SSIM	FR	0.8399	0.8877	0.6433	8.8229
**QWT-IQA**	**FR**	**0.9003**	**0.9262**	**0.7225**	**7.2260**
SISBLIM	NR	0.8749	0.8722	0.6926	9.3734
DIIVINE	NR	0.7080	0.7458	0.5144	12.8032
BLIINDS-II	NR	0.6156	0.6437	0.4465	14.6627
NIQE	NR	0.8708	0.9093	0.6841	7.9719

**Table 2:** Several IQA algorithm comparison on the blur and noise image dataset.

IQA metrics	Type	SROCC	PLCC	KROCC	RMSE
PSNR	FR	0.7088	0.7752	0.5290	11.7869
SSIM	FR	0.7023	0.7745	0.5251	11.7999
FSIM	FR	0.8644	0.8805	0.6700	8.8417
MS-SSIM	FR	0.8629	0.8914	0.6754	8.4553
**QWT-IQA**	**FR**	**0.9058**	**0.9174**	**0.7291**	**7.4238**
SISBLIM	NR	0.8793	0.8916	0.6956	8.4489
DIIVINE	NR	0.6021	0.6902	0.4363	13.5067
BLIINDS-II	NR	0.0911	0.2895	0.0566	17.8559
NIQE	NR	0.7945	0.8483	0.6057	9.8792

**Table 3:** Several IQA comparison on the LIVEMD image database.

IQA metrics	Type	SROCC	PLCC	KROCC	RMSE
PSNR	FR	0.6771	0.7398	0.5003	12.7237
SSIM	FR	0.6459	0.7333	0.4633	12.8388
FSIM	FR	0.8637	0.8932	0.6729	8.5048
MS-SSIM	FR	0.8392	0.8749	0.6474	9.1596
**QWT-IQA**	**FR**	**0.9043**	**0.9203**	**0.7294**	**7.4036**
SISBLIM	NR	0.8776	0.8952	0.6916	8.4303
DIIVINE	NR	0.6563	0.7183	0.4778	13.1586
BLIINDS-II	NR	0.1774	0.3895	0.1287	17.4188
NIQE	NR	0.7725	0.8377	0.5796	10.3299

**Fig 5:**
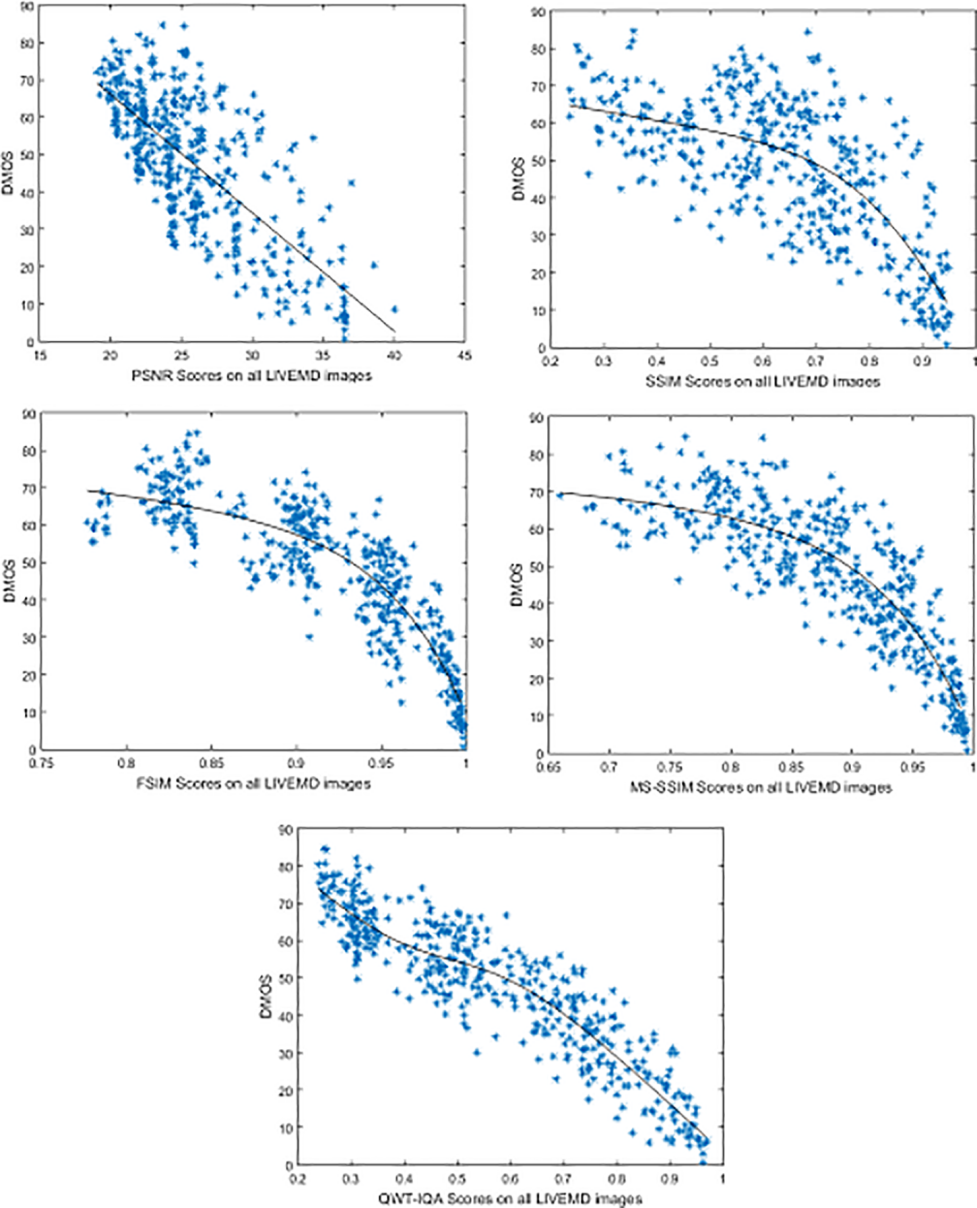
QWT-IQA scores against DMOS on the LIVEMD image database.

We also give the scatter plot of several FR IQA scores on the LIVEMD image database against DMOS in Fig 5, which also shows the QWT-IQA algorithm has a better agreement with the human subjective perception than PSNR, SSIM, FSIM and MS-SSIM.

### Experiment on the MDID2013 image database

Multiply distorted image database (MDID2013) [16] consists of 12 reference images and 324 distorted images simultaneously distorted by JPEG compression, blurring and noise injection. One half of pristine images of size 768×512 are from Kodak database, and the other half pristine images of size 1280×720 from LIVEMD database. The difference mean opinion score (DMOS) of MDID2013 is between 0 and 1.

The comparison results of FR QWT-IQA, PSNR, SSIM, FSIM and MS-SSIM are given in Table 4, as we can see from Table 4, our proposed QWT-IQA algorithm significantly outperforms these FR IQA algorithms on all cases.

**Table 4:** Several IQA comparison on the MDID2013 image database.

IQA metrics	Type	SROCC	PLCC	KROCC	RMSE
PSNR	FR	0.5604	0.5507	0.3935	0.0421
SSIM	FR	0.4494	0.4570	0.3143	0.0452
FSIM	FR	0.6431	0.6500	0.5314	0.0389
MS-SSIM	FR	0.7401	0.7435	0.5418	0.0340
**QWT-IQA**	**FR**	**0.7794**	**0.7896**	**0.5617**	**0.0312**
**SISBLIM**	**NR**	**0.8079**	**0.8140**	**0.6146**	**0.0295**
DIIVINE	NR	0.4463	0.4471	0.3644	0.0455
BLIINDS-II	NR	0.1796	0.2244	0.1200	0.0495
NIQE	NR	0.5450	0.5635	0.3787	0.0420

We also compare the QWT-SIM metric with some NR metrics such as SISBLIM, DIIVINE, BLIINDS-II and NIQE, listed in Table 4. It can be seen our QWT-SIM is a little inferior to NR SISBLIM, but NR SISBLIM is test on only 20% test images of database, so it is unfair comparison.

**Fig 6:**
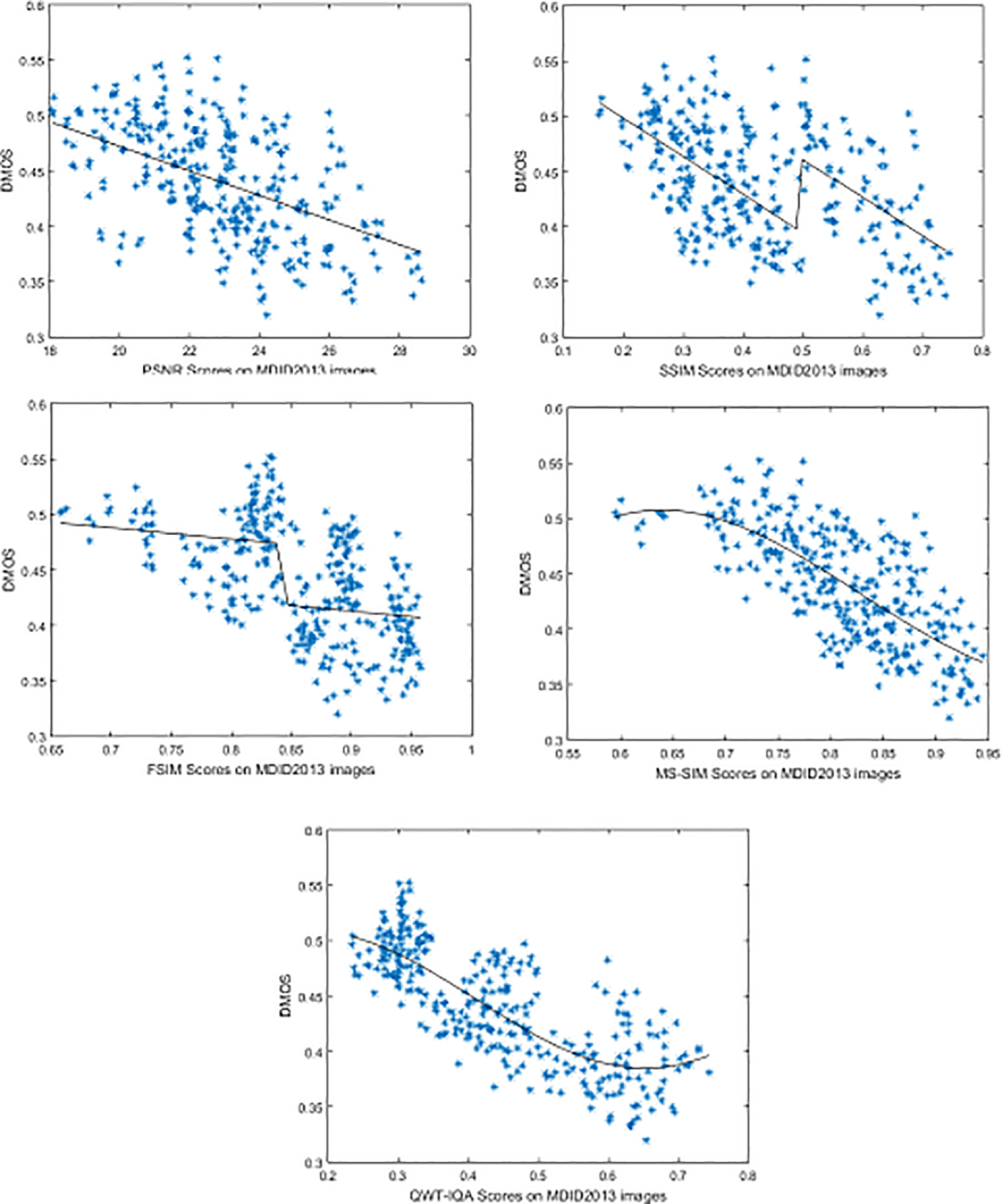
Scatter plots of several FR IQA algorithms on the MDID2013 image database.

On the MDID2013 image database, we also plot the scatter of QWT-IQA scores against DMOS, as shown in Fig 6. As seen clearly from Fig 6, the QWT-IQA algorithm has a good agreement with human subjective perception. This conclusion is consistent with that drawn from the previous experiment in LIVEMD database. In a word, many results have demonstrated that our method is a good technique for IQA.

### Experiment on the single distortion LIVE IQA database

LIVE IQA database [17] is single distortion database, which consists of 29 reference images and all 982 images, including five different distortion categories: JPEG2000 (JP2K) and JPEG compression, white Gaussian noise (WN), Gaussian blur (blur) and a Rayleigh fast fading channel distortion (FF).

For further test our proposed QWT-IQA metric, we also compare several IQA algorithms on the LIVE single distortion IQA database, and the results are listed in Table 5. It can be seen our QWT-IQA is only a little inferior to FSIM, and still gets good linear relationship with the human subjective scores.

**Table 5:** Several IQA comparison on single distortion LIVE image database.

IQA metrics	Type	SROCC	PLCC	KROCC	RMSE
PSNR	FR	0.8756	0.8723	0.6865	13.3597
SSIM	FR	0.9479	0.9449	0.7963	8.9455
**FSIM**	**FR**	**0.9634**	**0.9597**	**0.8337**	**7.6781**
MS-SSIM	FR	0.9513	0.9489	0.8045	8.6187
**QWT-IQA**	**FR**	**0.9517**	**0.9562**	**0.8159**	**6.7638**
SISBLIM	NR	0.9450	0.9505	0.7981	8.5136
DIIVINE	NR	0.8304	0.8217	0.6856	15.614
BLIINDS-II	NR	0.9067	0.9143	0.7369	11.096
NIQE	NR	0.9236	0.9162	0.7546	10.982

### Time efficiency of IQA metrics

Time efficiency is another important index for algorithm. We give the time of compared algorithm listed in Table 6. It can be seen most of the NR methods need more time to train images, and the FR methods only need to compute the relevance or deviation in a very short time. Our proposed QWT-IQA is more time consuming than FR PSNR, SSIM and MS-SSIM, but also efficient for real time application.

**Table 6:** Efficiency comparison of several IQA.

IQA metrics	Type	Times (s)
PSNR	FR	0.05
SSIM	FR	0.19
FSIM	FR	1.12
MS-SSIM	FR	0.33
QWT-IQA	FR	0.55
SISBLIM	NR	1.80
DIIVINE	NR	25.40
BLIINDS-II	NR	76.12
NIQE	NR	0.32

## Conclusion

In this paper, we have proposed a QWT-based full reference IQA metric called QWT-IQA for multiply distorted images. It first calculates the local similarity of phase and magnitude of each subband via QWT, and then uses a weighting method to gain image quality score through considering human visual characteristics. Many experimental results have demonstrated that our QWT-IQA has a higher consistency with the subjective measurement on multiply distortion images, compared with the state-of-the-art full reference IQA methods.
